# Climate Change and Biotic Interactions Will Change the Distributions of Ungulates on the Qinghai–Tibet Plateau

**DOI:** 10.3390/ani16020183

**Published:** 2026-01-08

**Authors:** Tong Zhang, Yikai Wang, Fu Shu, Yonglei Lv, Zijun Tang, Feng Liu, Zhiguo Li, Yuan Wang, Guangwei Tang, Guanglong Wang, Nanfei Wu, Keji Guo, Xumao Zhao

**Affiliations:** 1 Central South Academy of Inventory and Planning of National Forestry and Grassland Administration, Changsha 410014, China; 2 College of Ecology, Lanzhou University, Lanzhou 730000, China; 3 Forestry Survey and Planning Research Institute of Xizang Autonomous Region, Lhasa 850000, China; 4 Key Laboratory of Zoological Systematics and Evolution, Institute of Zoology, Chinese Academy of Sciences, Beijing 100101, China; 5 University of Chinese Academy of Sciences, Beijing 100049, China; 6 College of Life-science and Technology, Central South University of Forestry and Technology, Changsha 410000, China; 7 College of Life Sciences, Hunan Normal University, Changsha 410000, China

**Keywords:** species distributions, conservation, species interactions, range shift

## Abstract

Species interactions are crucial for understanding how species will respond to future climate change. Incorporating interspecific relationships into mammalian distribution prediction models will significantly impact model outcomes, especially those for animals on the Qinghai–Tibet Plateau (QTP). Thus, we incorporated interspecific relationships into species distribution models to assess and predict the future distributions of five ungulates, including the Red deer (*Cervus elaphus*), the Kiang (*Equus kiang*), the Tibetan gazelle (*Procapra picticaudata*), the Tibetan antelope (*Pantholops hodgsonii*), and the Bharal (*Pseudois nayaur*). We found that (1) the suitable habitats of these five ungulates were all predicted to increase between the present and 2050; (2) the suitable distribution areas of four of these ungulates were predicted to be smaller when interspecific relationships were incorporated into the models, with the exception of the Red deer, whose suitable habitat was estimated to be larger; and (3) the centroids of suitable habitat for the five ungulates were predicted to shift to the southern part of the QTP by 2050. Our results demonstrated that interspecific relationships could influence predictions of species distributions, and thus incorporating interspecific relationships will facilitate better assessments and predictions of the future distributions of species.

## Simple Summary

In this study, we use species distribution models (SDM) to predict suitable habitats of five ungulate species on the Qinghai–Tibet Plateau (QTP) and predict the impacts of climate change and human activities on their future habitat change. Also, we incorporated interspecies interactions into the models. Study shows that including biotic factors significantly changes prediction results, indicating that future conservation measures should consider more about interspecies interactions.

## 1. Introduction

The Qinghai–Tibet Plateau (QTP), often referred to as the world’s “third pole”, plays an important role in the Earth’s climate system, maintaining the ecological security of the northern hemisphere [1,2]. The QTP is experiencing unprecedented climate change [3], with average temperatures rising by up to 0.3 °C per decade, about three times the rate of global warming [1]. These rapid changes have made the ecosystems on the QTP particularly fragile [3]. In fact, many ecological properties on the QTP, including phenology, diversity, biomass, interspecific relationships, and the distributions of species, have suffered from the impacts of climate change over the past few decades [4,5,6,7,8,9].

Species distribution models (SDMs) use the occurrences of species along with actual environmental data to estimate the ecological niches of target species according to a specific mathematical algorithm [10]. Predictions of species distributions are mainly determined by abiotic factors and biotic interactions [11,12]. A growing body of evidence points to the importance of species interactions in determining the geographical ranges of species [13]. Moreover, with climate change, interspecific interactions will also be reshaped [14,15]. When predicting the distributions of multiple species in sympatry under future climate conditions, the interrelationships between multiple species will significantly influence the outcome of the predictions [16]. Studies have provided certain methodologies, but these are not applicable to terrestrial ungulates lacking specific symbiotic relationships [16,17].

Ungulates on the QTP are crucial to maintaining the ecological security of the plateau via their positions in the food chain [18]. Ungulate habitats are particularly vulnerable to climate change, as climate-driven habitat changes may lead to the disproportionate loss of key connectivity between ungulate habitats [19]. However, the declines in ungulate habitats on the QTP have been much more severe than elsewhere in the world [7]. Studies have found that the many ungulate species on the QTP are predicted to endure habitat loss as well as shifts in their ranges to higher latitudes [20,21]. These changes have affected not only the composition and diversity of local ecosystems but have also changed species interactions [12]. Therefore, the responses of ungulates to climate change are of great significance for the effective protection of biodiversity and maintaining the integrity of ecosystem functions on the QTP [22].

Forty percent of China’s ungulates are found on the QTP [23], of which the Red deer (*Cervus elaphus*), the Kiang (*Equus kiang*), the Tibetan gazelle (*Procapra picticaudata*), the Tibetan antelope (*Pantholops hodgsonii*), and the Bharal (*Pseudois nayaur*) play the most significant roles in the stability of ecosystems by supporting carnivore populations [23]. *Equus kiang*, *Procapra picticaudata*, and *Pantholops hodgsonii* are endemic to the QTP, and *Cervus elaphus* and *Pseudois nayaur* are important species on the QTP [24,25,26,27,28,29]. The Tibetan antelope and Tibetan gazelle are typical plateau animals, living in alpine meadows, grasslands, alpine deserts, and intermountain valleys below an altitude of 5000 m [25,26,30]. The Bharal is one of the most widely distributed ungulates on the QTP and inhabits mountainous bare rock areas and valley meadows at an altitude of 500–2500 m [27,31]. The Kiang is endemic to the QTP and inhabits plateau steppe, alpine desert steppe, and mountain desert areas at an altitude of 2700–5200 m [31]. The Red deer consists of eight subspecies in China, which are widely distributed in China, and are classified as national secondary key protected wild animals [32].

The sensitivity of ungulates on the QTP to climate change as well as existing competition among different ungulates [21,33,34] both have impacts on their distributions. In this study we aim to (1) consider the impact of biotic and abiotic factors on distributions of ungulate species on the QTP and (2) predict future changes in the distributions of these five ungulate species on the QTP and identify conservation priorities.

## 2. Methods

### 2.1. Data Collection

**Figure 1:**
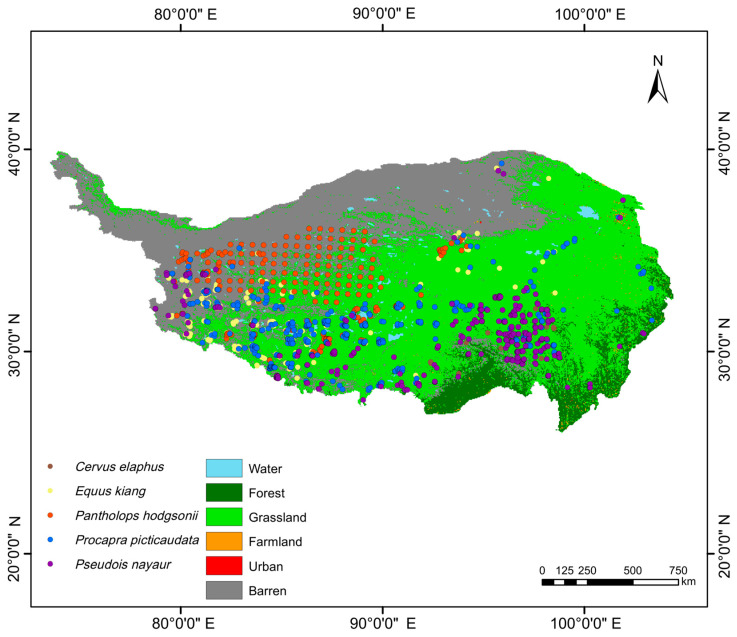
Occurrences of *Cervus elaphus*, *Equus kiang*, *Pantholops hodgsonii*, *Procapra picticaudata*, *Pseudois nayaur* within the study area on the Qinghai–Tibet Plateau.

We collected occurrence data for the Red deer, the Kiang, the Tibetan gazelle, the Tibetan antelope, and the Bharal on the QTP using field investigations, the GBIF database [35], and the National Qinghai–Tibet Plateau Scientific Data Center [36]. We investigated distributions of ungulates on the QTP from 2019 to 2024 using both the line transect method. We recorded the species and locations of ungulates along the road from off-road vehicles. From July in 2022 to September in 2024, we laid out 450 transects, each range from 1.28 km to 43.41 km, with an average of 5.62 km, and the total length is 2531.73 km (Figure 1). Approximately 30 people participated in the field investigation of this project in groups. In the sample line survey two observers and a driver traveled slowly (20–40 km/h) in a 4 × 4 vehicle. Observers scanned both sides of the road with 8 × 30 binoculars, and recorded GPS coordinates. We recorded the locations where the actual entities, traces, and feces of the animals appeared. To exclude the influence of spatial autocorrelation on species distribution simulations and predictions, we used the “spThin” package in R to screen distribution point data [37]. Occurrences for the five ungulates were retained only if they fell within 5 km of their known ranges.

We obtained bioclimate variables data from the CHELSA database, a high-resolution climate database of the Earth’s land surface regions [38]. The current data (spatial 30 arc-seconds resolution) was the average of meteorological data obtained from meteorological stations between 1979 and 2013 [38]. We derived future bioclimatic variables (2050) from Intercomparison Project Phase 6 (CMIP6) (https://worldclim.org/data/cmip6/cmip6climate.html) (accessed on 15 March 2024). In consideration of the geographical environment of China, we selected the BCC-CSM2-MR climate model under CMIP6 and the SSP245 climate scenario data under Shared Socio-Economic Pathways (SSPs). SSP2-4.5 represents a moderate and robust future scenario [39]. The BCC-CSM2-MR model is a coupled climate model developed by the Beijing Climate Center, which performs well in the tropospheric temperature and circulation in the East Asian and Indian monsoon regions [40]. In addition, we obtained elevation data from the Gridded Bathymetry Data (https://www.gebco.net/data_and_products/gridded_bathymetry_data/#global) (accessed on 12 March 2024) and land cover data from GeoSOS (https://geosimulation.cn) (accessed on 18 March 2024), which have both been shown to affect the distributions of species. We also incorporated other terrain factors, including aspect and slope. We calculated these data based on elevation data by ArcMap 10.8.

### 2.2. MaxEnt Models

We simulated the present and future distributions of the five ungulates using the MaxEnt model. The MaxEnt model can simulate the complex nonlinear relationship between the response variable and the predictor variable based on the maximum entropy machine learning algorithm [41], which can also produce good predictions from small samples [42]. To avoid overfitting, we excluded collinearity between bioclimatic variables [43]. If the collinearity value |r| between the two variables was >0.7 (Figure S1), we retained the more important one [44].

We used the “ENMeval” package and the “dismo” package in R3.42 to run MaxEnt models [45,46]. To optimize performance of the MaxEnt model, we set feature classes (FC) and the regularization multiplier (RM), which have important influences on the performance of the model [10]. We used three types of features, including linear (L), quadratic (Q), and hinge (H), as well as combinations among them, and set eight values of RM (0.5, 1, 1.5, 2, 2.5, 3, 3.5, and 4). We used combinations of features and RM, producing 32 models using 10,000 randomly selected background points in the study area [47].

We divided the occurrence data into four “blocks” by maximum and minimum latitude and longitude, with one block for testing and three blocks for training, repeating this process until all blocks had been used for testing [47]. Then, we selected the best combination of the FC and RM models among the 32 models [9]. First, we selected the model with the lowest average omission rate using a 10% omission rate (OR10), which is the probability of a test occurrence whose predicted fitness was lower than 10% of the training value. If multiple models had the same OR10, we retained the model with the highest average validation AUC. We also used the Boyce index to reflect the quality of the model, which has been shown to be a reliable measure based on the prediction of species with only distribution points [41]. The values of the Boyce index ranged from −1 to 1, with a value greater than 0 indicating that the predicted range of the species was consistent with the actual distribution [41].

Using the cloglog transform of the MaxEnt model to output the prediction results [48], a 10% training presence cloglog threshold was used to convert the habitat predicted by the model into a binary habitat map with a value of 0 or 1, with habitat less than this threshold assigned a value of 0, indicating unsuitable habitat. Habitat greater than this threshold was assigned a value of 1, which represents suitable habitat. Finally, the current and future suitable habitat distribution maps of the five ungulates were obtained, and changes in their distributions were estimated [20]. Based on the dichotomous map, we calculated the geographic center of each species’ habitat, i.e., the centroids. The migrations of the centroids over time reflects the migration of species under the impacts of climate change and human activities [49].

We defined two models, the abiotic-only model and the abiotic–biotic model. The abiotic-only model included abiotic factors, and the abiotic–biotic model included both abiotic factors and biotic factors. We incorporated biotic factors into predictions of species distributions [50]. For each ungulate, we added the results of the abiotic model as environmental factors to the species distribution model, with the distributions of the four other ungulates as predictors [51]. For example, to consider the influence of the interspecific relationship between the Tibetan antelope and the Tibetan gazelle on their distributions, we regarded the distribution of the Tibetan antelope simulated by abiotic-only models as an environmental factor for the distribution of the Tibetan gazelle, and so on.

## 3. Results

### 3.1. Effects of Climate Change on the Distributions of Five Ungulates

Our field investigations indicated that the five ungulates under investigation have sympatric distributions on the QTP (Figure 1). We obtained models and the response curves for each model (Figure S2). Models for all five species had high AUC values (above 0.8) and high continuous Boyce index (CBI) values (above 0.9), indicating that the predicted distributions were consistent with present data (Table 1). After screening, the optimal 10 models for all five species under both abiotic and biotic conditions were obtained. The CBI index of each of these models was greater than 0.9, and the AUC value of each was greater than 0.8, indicating that these models performed significantly better than the null model.

**Figure 2:**
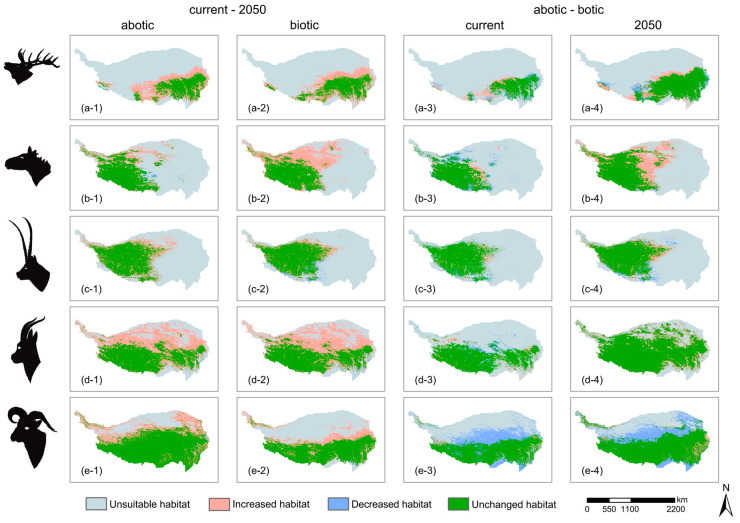
Habitat shifts of each species across different scenarios, (**a**) *Cervus canadensis*, (**b**) *Equus kiang*, (**c**) *Pantholops hodgsonii*, (**d**) *Procapra picticaudata*, (**e**) *Pseudois nayaur*, 1–4 of each species represent different comparisons, showing habitat ranges that increase, decrease, or remain unchanged in the latter scenario relative to the former: 1: current–2050 (abiotic); 2: current–2050 (biotic); 3: current (abiotic)–current (biotic); 4: 2050 (abiotic)–2050 (biotic).

Under abiotic-only models, the suitable habitats of all species were predicted to increase from 2000 to 2050, of which the suitable habitat area of Red deer was predicted to increase by 87.69%, followed by the Tibetan gazelle (64.67%), the Bharal (23.49%), the Kiang (12.37%), and the Tibetan antelope (11.70%) (Table 2, Figure 2 and Figure S3). Under abiotic-only models, the centroids of suitable habitat from 2000 to 2050 for the five ungulates were predicted to shift 592 km to the southeast for the Red deer, 847 km to the southwest for the Kiang, 751 km to the northwest for the Tibetan antelope, 292 km to the southwest for the Tibetan gazelle, and 427 km to the southeast for the Bharal (Table 3).

**Figure 3:**
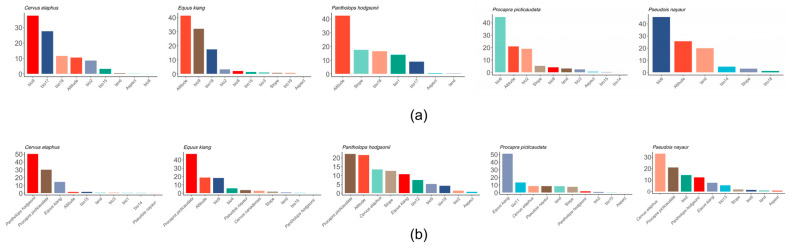
Permutation importance of all variables for each species under (**a**) abiotic-only models and (**b**) abiotic–biotic models.

Among the many factors that affect the distributions of species, bio9 (average temperature in the driest quarter) and bio17 (precipitation in the driest quarter) had the greatest influence on the Red deer, altitude and bio9 had the greatest influence on the Kiang, altitude and slope had the greatest influence on the Tibetan antelope, bio6 (minimum temperature in the coldest month) and altitude had the greatest influence on the Tibetan antelope, and bio6 and altitude had the greatest influence on the Bharal (Figure 3).

### 3.2. Effects of Interspecific Relationships on the Distributions of Five Ungulates

Under abiotic–biotic models, the suitable habitat of the Kiang was predicted to increase by 67.13% from 2000 to 2050, followed by the Tibetan gazelle (66.14%), the Red deer (64.69%), the Bharal (27.28%), and the Tibetan antelope (20.42%).

Compared with the abiotic model, the distribution of the Red deer was predicted to be 6% larger under the abiotic–biotic model, while the distributions of the other ungulates were predicted to be smaller. Among them, the estimated distribution of the Bharal was 36% smaller, that of the Kiang was 6% smaller, that of the Tibetan gazelle was 2% smaller, and the distribution of the Tibetan antelope was very slightly decreased (Table 2, Figure 2 and Figure S4).

Under abiotic–biotic models, the centroids of suitable habitat for the five ungulates from 2000 to 2050 were predicted to shift 560 km to the southeast for Red deer, 714 km to the southwest for the Kiang, 757 km to the southwest for the Tibetan antelope, 296 km to the southwest for the Tibetan antelope, and 417 km to the southeast for the Bharal (Table 3).

Compared with the abiotic-only models, the shifts in the distributions of the Tibetan antelope and the Tibetan gazelle were predicted to be larger under abiotic–biotic models, while the shifts in the distributions of the Red deer, the Kiang, and the Bharal were predicted to be smaller.

## 4. Discussion

Climate change not only affects the distributions of species but also influences their interspecific relationships, thereby posing challenges to the conservation of species diversity [52]. We evaluated and predicted the distribution changes of five ungulates (the Red deer, the Kiang, the Tibetan gazelle, the Tibetan antelope, and the Bharal) on the QTP using species distribution models, incorporating intra-species factors to predict distribution shifts of these five ungulates. We found that under likely future climate conditions, the distributions of these five ungulates were all predicted to increase significantly, with relationships between species impacting their distributions (Table 2 and Table 3).

### 4.1. The Distributions of Five Ungulates on the QTP Were Predicted to Expand Under Future Climate Change

We found that the distributions of the five ungulate species were predicted to increase significantly under future climate conditions (Figure 2). Spatial differences in vegetation changes on the Qinghai–Tibet Plateau have been caused by climate change [53]. Over the past 40 years, the warming and humidification of the Qinghai–Tibet Plateau has led to an extension of the growing season of the dominant species in alpine meadows [54]. Based on climate change predictions, the coverages of alpine meadows, alpine grasslands, and temperate grasslands on the Qinghai–Tibet Plateau have all been predicted to significantly increase by 2060 [55], and the proportion of alpine meadows in grasslands and the dominance of *Kobresia* and *Stipa* communities have both increased over the past 40 years [54]. These changes in vegetation likely underlie the predicted increases in the distributions of the five species of ungulates under investigation here.

Research has also shown that, from the 1980s to the 2020s, the ranges of mammals, reptiles, and amphibians have expanded because of climate change [56]. In recent years, some areas have experienced excessive expansion of herbivore populations due to climate change [57,58]. Our results are consistent with the trend of increasing mammal distributions over the past 40 years (1980–2020) [56].

However, due to the heterogeneity of the Qinghai–Tibet Plateau, the vegetation coverage varies in different regions. Water has been shown to be the dominant factor in the change of vegetation in the northwest QTP, making the vegetation in this region green with the increase of precipitation, and temperature was the main factor affecting vegetation growth in relatively humid areas [53]. We also found that bio9 (average temperature in the driest quarter), bio17 (precipitation in the driest quarter), and bio6 (minimum temperature in the coldest month) were the most important factors underlying predicted increases in the distributions of four ungulates (Figure 3). Temperature and precipitation can directly affect the behaviors and physiological tolerances of species and indirectly affect the growth of vegetation that provides food for species [59]. Warmer temperatures and increased rainfall have led to changes in vegetation, which may change the sizes of habitats and may ultimately lead to the expansion of ungulates. By affecting reproductive behaviors, climate change poses a significant impact to species’ survival [60]. Such behavioral shifts can directly change population trajectory [61].

### 4.2. The Distributions of Ungulates Were Predicted to Be Smaller When Considering Biotic Variables

Climate change will lead to dramatic changes in the diversity patterns of ungulates on the Qinghai–Tibet Plateau, which will affect the stability of the entire ecosystem [62]. Interspecific relationships will also affect the distribution of species under the future climate change [16,63]. There is an overlap in ecological niches among these ungulates [24,25,26,28,64], which leads to at least some competition between species. When considering the relationships among species, the distributions of the Bharal, the Kiang, the Tibetan gazelle, and the Tibetan antelope were predicted to be reduced in size (Table 2). The Tibetan antelope, the Kiang, and the Tibetan gazelle have similar foods, and their dietary were estimated to overlap between 0.785 and 0.869 [65]. Gramineous plants are the major food of three of these herbivores, accounting for 58.7% of the diet of the Tibetan antelope, 44.57% for the Tibetan gazelle, and 92.28% for the Kiang [65].

Among these ungulates, the range of the Red deer was predicted to be 6% larger when considering both biotic and abiotic factors, while that of the Bharal was predicted to be 35% smaller (Table 2). It showed that interspecific interactions had the greatest impact on the Bharal compared to the other studied species (Figure 3). The Bharal and the Red deer have similar ecological needs and share common geographic distributions [64,66]. Compared to the Red deer, the Bharal has a smaller range than the Tibetan gazelle or the Tibetan antelope and a less diverse habitat than the Bharal [28,66,67,68]. Research indicates that as the number of co-occurring species increases, the ecological niche width of the Bharal decreases, which suggests that competition with other species hinders the Bharal from occupying all suitable habitats [67].

### 4.3. Implications for Conservation

Under the influence of climate change, biodiversity is undergoing re-distributions both in time and space, which necessitates the establishment of new mechanisms for biodiversity conservation and management [55]. The predicted shifts in the distributions of ungulates suggest that the establishment and prioritization of future protected areas should follow the migrations of these species. At the same time, migrating species may compete with species in other areas, requiring continuous observation. The increase in the number of Tibetan antelope in recent years is attributed to the establishment of new national protected areas [68], which provides lessons for future conservation efforts. Though currently listed as Least Concern (LC) with an optimistic conservation status [27], the Bharal faces potential habitat constraints due to intensifying competition [67]. Improving landscape connectivity or establishing species migration corridors can help species adapt to environmental changes more effectively and quickly, thereby alleviating the pressures brought about by climate change [62].

To track future climate changes, the core distribution areas of these five ungulates need to be shifted between 200 and 800 km to the southeast and southwest of the Qinghai–Tibet Plateau. The distribution ranges of Red deer and Kiang are projected to expand into new areas in the northern and eastern regions (Figure 2). In addition to preemptively protecting the future suitable habitats of these species, corridors that connect the current and future suitable habitats should be constructed. The impact of discontinuous suitable ranges on migration should also be incorporated into conservation planning. For the Red deer, corridors must be established in the southern QTP to connect fragmented suitable ranges in the west with those in the east (Figure 2). This is necessary to minimize the interference from human activities on the migration and diffusion paths as much as possible. Under the future climate change conditions, interspecific relationships will also affect the distribution of species [16,69]. Therefore, when considering how the ungulates on the Qinghai–Tibet Plateau respond to climate change, the influences of interspecific relationships should also be considered.

## 5. Conclusions

We demonstrate that climate change is predicted to drive significant range expansions for five ungulate species, and incorporating biotic variables may lead to shifts in species distribution ranges. These findings highlight the necessity of integrating interspecific relationships into species distribution modeling to improve predictive accuracy. In addition, other biotic pressures not included in the model may also influence the distributions of ungulates. For instance, predation pressure and human activities also affect interactions among species, which hindered the migration and dispersal of ungulates [70,71].

## Figures and Tables

**Table 1:** Optimal parameter settings for species distribution models. L: linear; Q: quadratic; and H: hinge, RM: regularization multiplier, AUC: Area Under Curve, CBI: Continuous Boyce Index.

Species	Models	Features	RM	Validation AUC	CBI	10% Omission Suitability Threshold
*Cervus elaphus*	abiotic	LQ	1.5	0.937	0.961	0.390
	biotic	L	2	0.934	0.928	0.264
*Equus kiang*	abiotic	LQH	4	0.894	0.972	0.211
	biotic	L	1	0.894	0.993	0.183
*Pantholops hodgsonii*	abiotic	L	1	0.844	0.970	0.386
	biotic	L	3.5	0.839	0.954	0.437
*Procapra picticaudata*	abiotic	LQ	4	0.835	0.996	0.311
	biotic	L	3.5	0.829	0.978	0.278
*Pseudois nayaur*	abiotic	L	4	0.801	0.926	0.285
	biotic	LQ	4	0.840	0.980	0.275

**Table 2:** Predictions of suitable habitats.

Species	Factors	2000/km^2^	2050/km^2^	Predicted Change by 2050
*Cervus elaphus*	abiotic	420,650	789,520	87.69%
	biotic	446,968	736,115	64.69%
*Equus kiang*	abiotic	706,687	794,130	12.37%
	biotic	661,144	1,104,968	67.12%
*Procapra picticaudata*	abiotic	980,647	1,614,829	64.67%
	biotic	971,945	1,614,829	66.14%
*Pantholops hodgsonii*	abiotic	779,818	871,049	11.70%
	biotic	779,068	938,176	20.42%
*Pseudois nayaur*	abiotic	1,486,542	1,835,705	23.49%
	biotic	955,793	1,216,531	27.28%

**Table 3:** Predicted migrations of species habitat centroids and changes in elevation.

Species	Factors	Predicted Direction of Migration by 2050	Predicted 2050 Migration Distance (km)	Current Elevation (m)	Predicted 2050 Elevation (m)
*Cervus elaphus*	abiotic	Southeast	591.922	5341	3718
	biotic	Southeast	560.279	5380	4746
*Equus kiang*	abiotic	Southwest	846.972	4719	5117
	biotic	Southwest	713.860	4532	4966
*Procapra picticaudata*	abiotic	Southwest	292.301	5039	5004
	biotic	Southwest	295.608	4743	5004
*Pantholops hodgsonii*	abiotic	Northwest	750.705	4616	5049
	biotic	Southwest	757.015	4721	5295
*Pseudois nayaur*	abiotic	Southeast	427.263	5062	4885
	biotic	Southeast	417.148	5132	4576

## Data Availability

The data are available upon reasonable request from the corresponding author.

## Supplementary Materials

The following supporting information can be downloaded at: https://www.mdpi.com/article/10.3390/ani16020183/s1, Figure S1: Correlations of bioclimatic variables; Figure S2: Response cruve of each species; Figure S3: Habitat suitability for each species (abotic): Suitable habitat for each species:(a) Cervus elaphus; (b) Equus kiang; (c) Pantholops hodgsonii; (d) Procapra picticaudata; (e) Pseudois nayaur; Figure S4: Habitat suitability for each species (abotic-biotic): Suitable habitat for each species: (a) Cervus elaphus; (b) Equus kiang; (c) Pantholops hodgsonii; (d) Procapra picticaudata; (e) Pseudois nayaur.
