# Supplementary material for: Climate Change and Biotic Interactions Will Change the Distributions of Ungulates on the Qinghai–Tibet Plateau
Source: Animals (Basel). 2026 Jan 8;16(2):183. doi: 10.3390/ani16020183 (PMC12838186; doi:10.3390/ani16020183)
Supplement: Supplementary file 1 [file animals-16-00183-s001.zip › animals-4025495-supplementary.pdf]

Supplementary materials

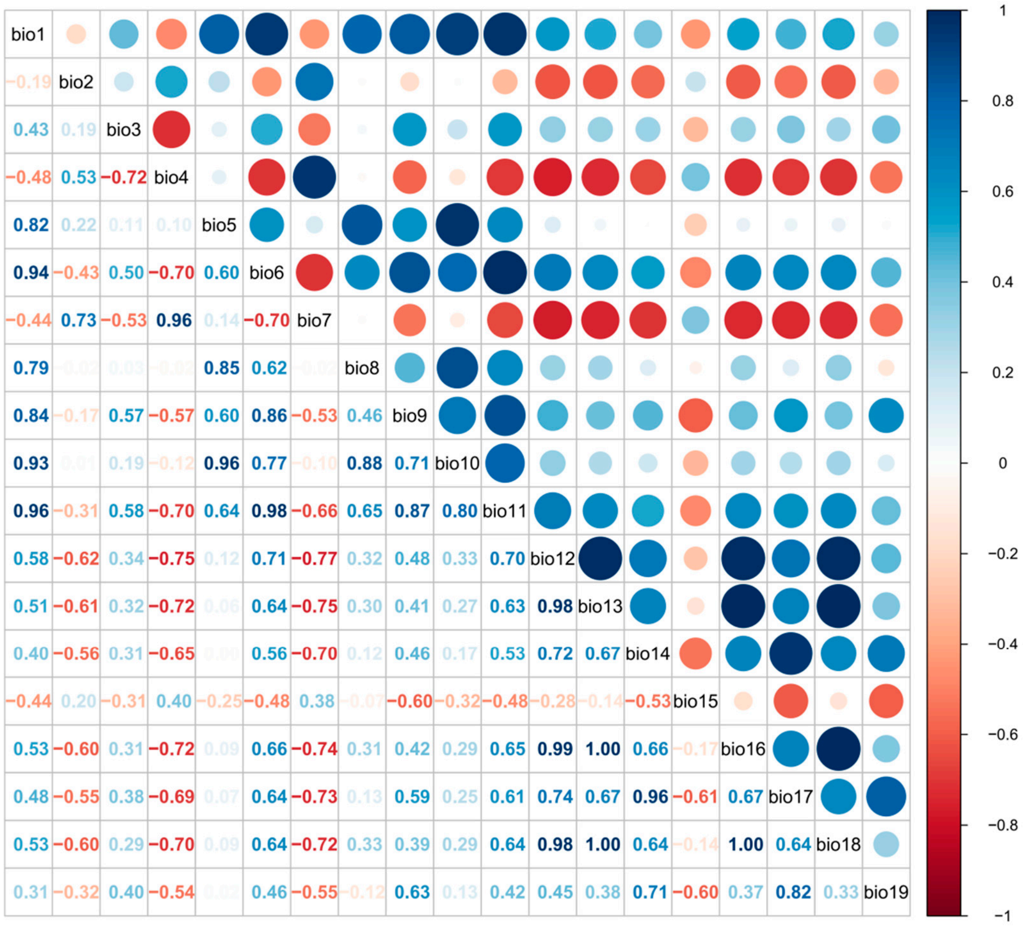

Figure S1. Correlations of bioclimatic variables.

Figure S2 Response curves of each species

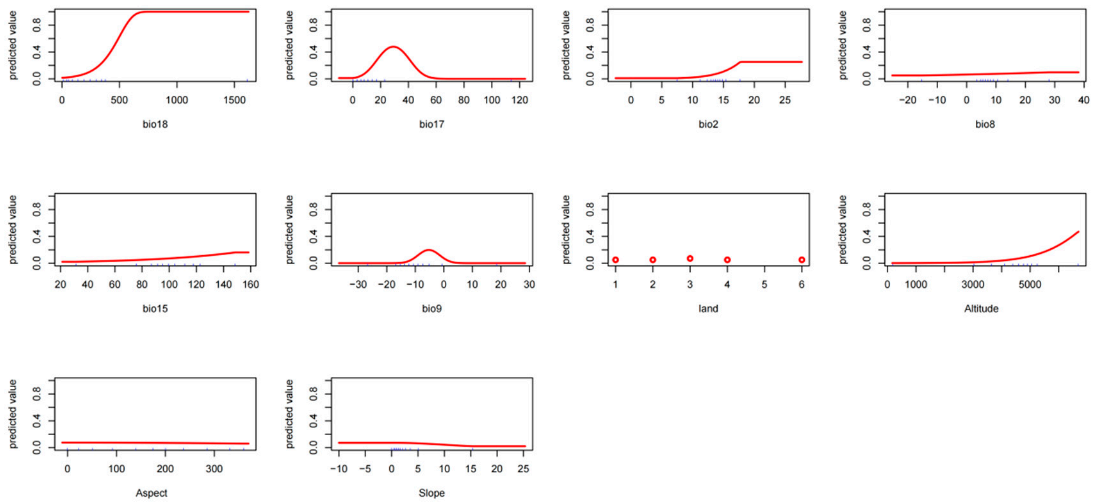

Figure S2-1. Response curves of *Cervus elaphus* (abotic only)

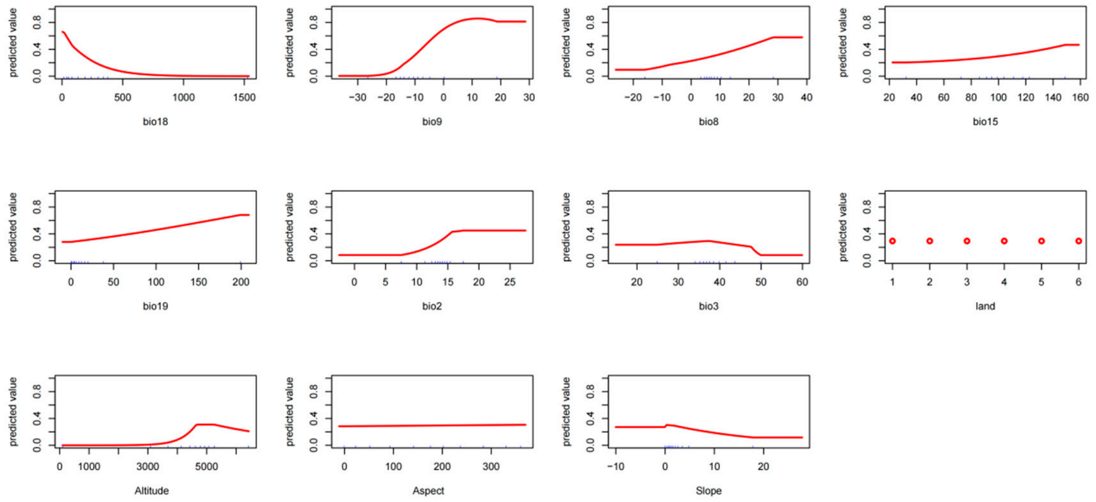

Figure S2-2. Response curves of *Equus kiang* (abotic only)

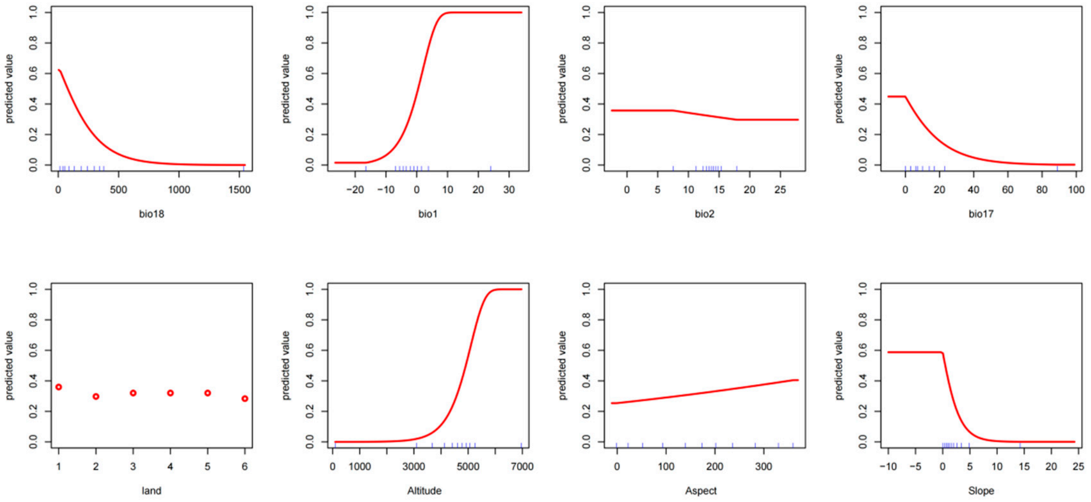

Figure S2-3. Response curves of *Pantholops hodgsonii* (abotic only)

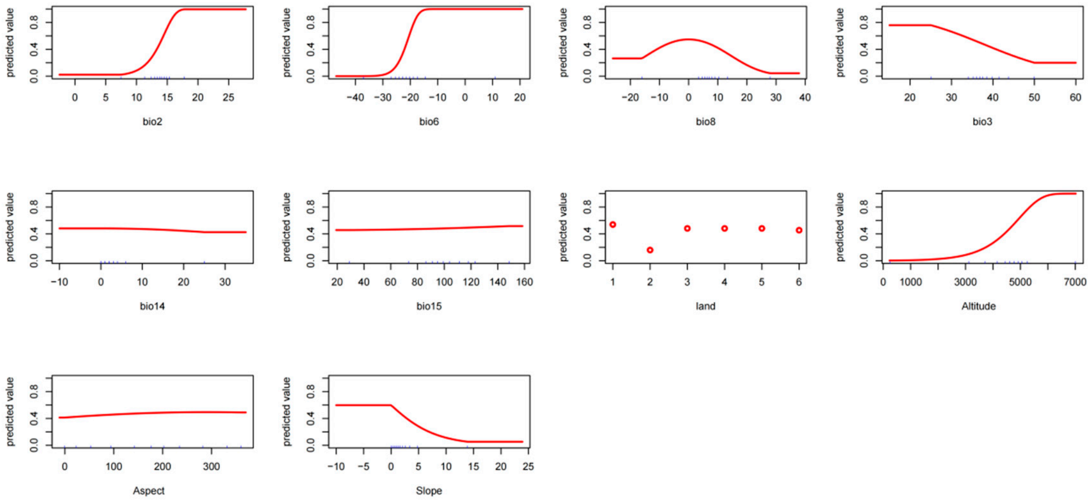

Figure S2-4. Response curves of *Procapra picticaudata* (abotic only)

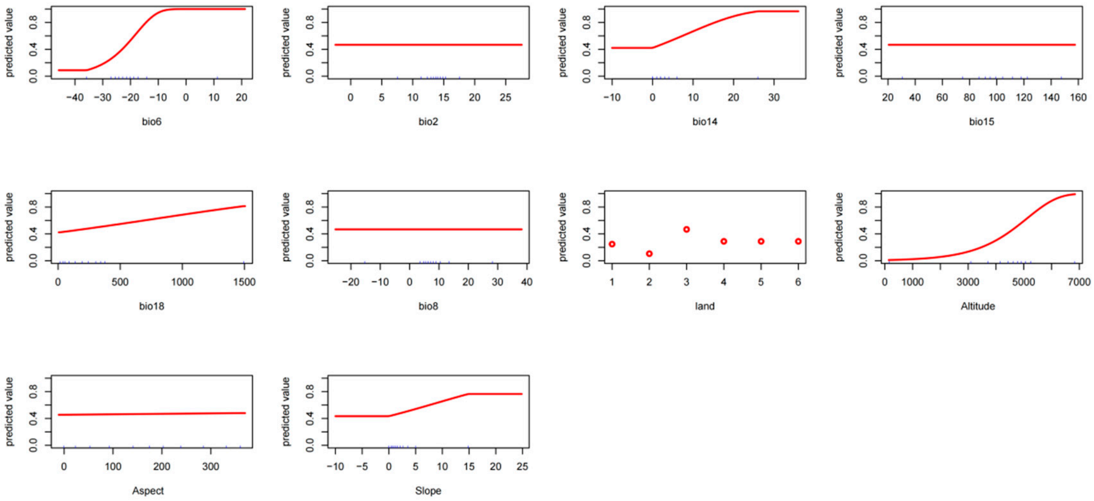

Figure S2-5. Response curves of *Pseudois nayaur* (abotic only)

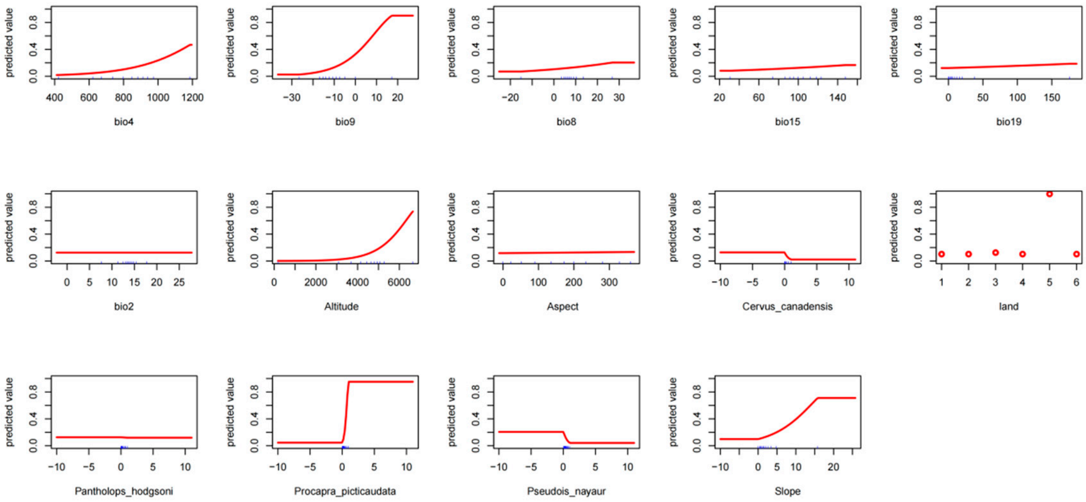

Figure S2-6. Response curves of *Cervus elaphus* (abotic-biotic)

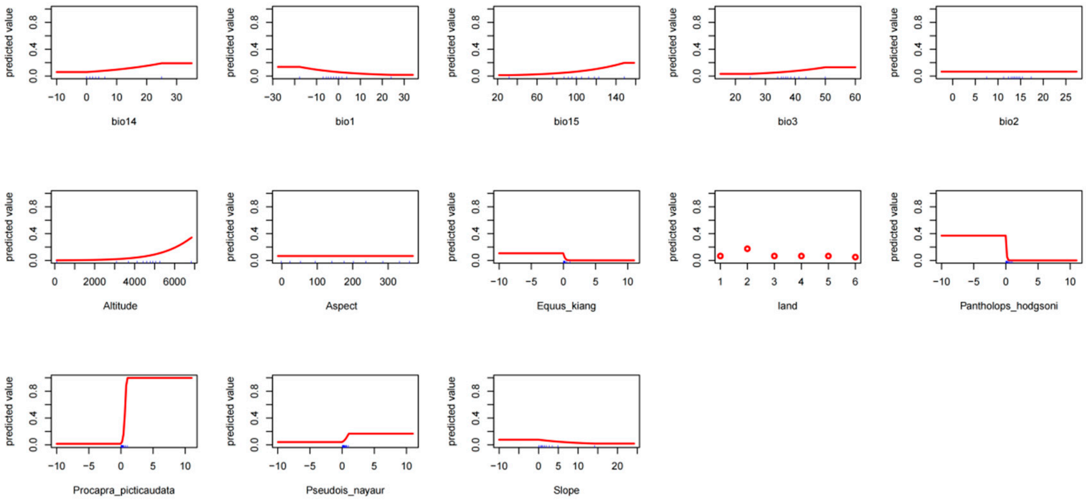

Figure S2-7. Response curves of *Equus kiang* (abotic-biotic)

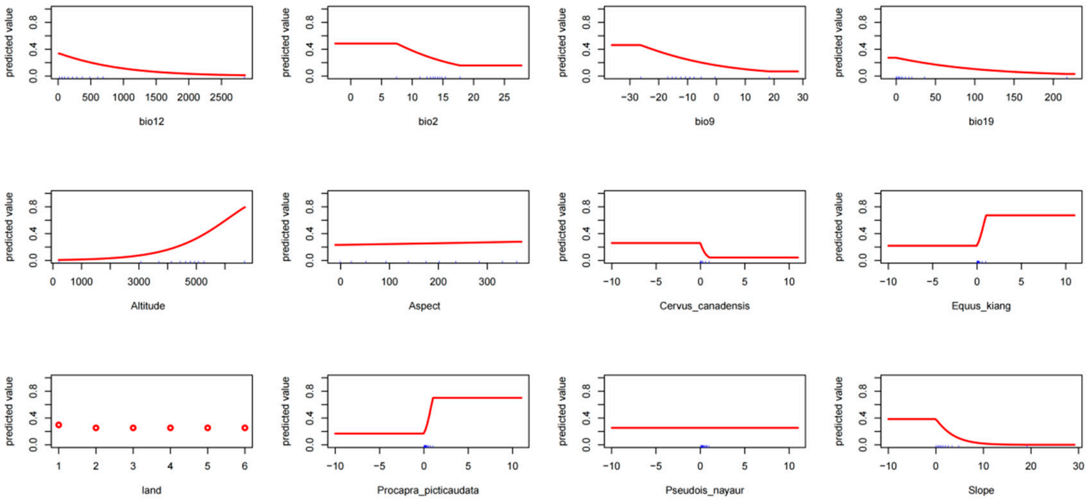

Figure S2-8. Response curves of *Pantholops hodgsonii* (abotic-biotic)

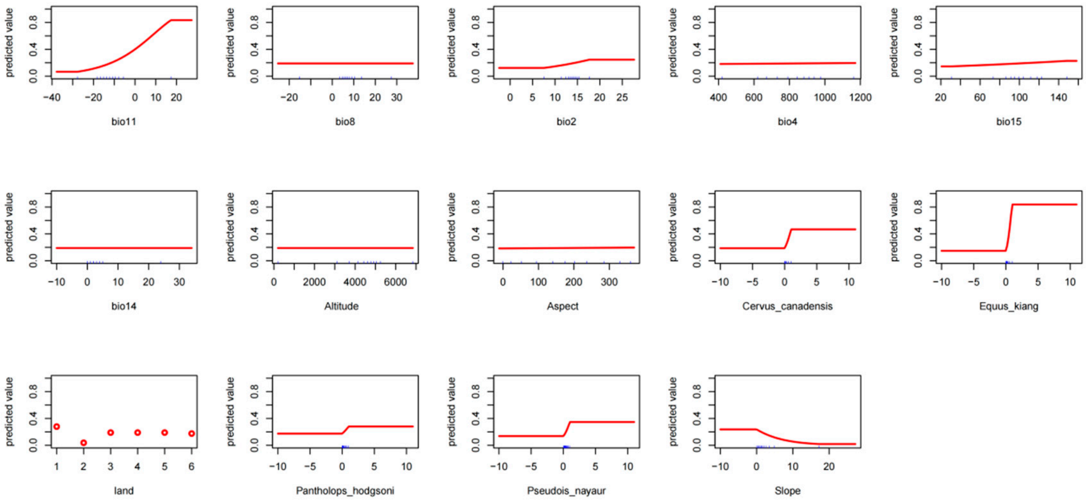

Figure S2-9. Response curves of *Procapra picticaudata* (abotic-biotic)

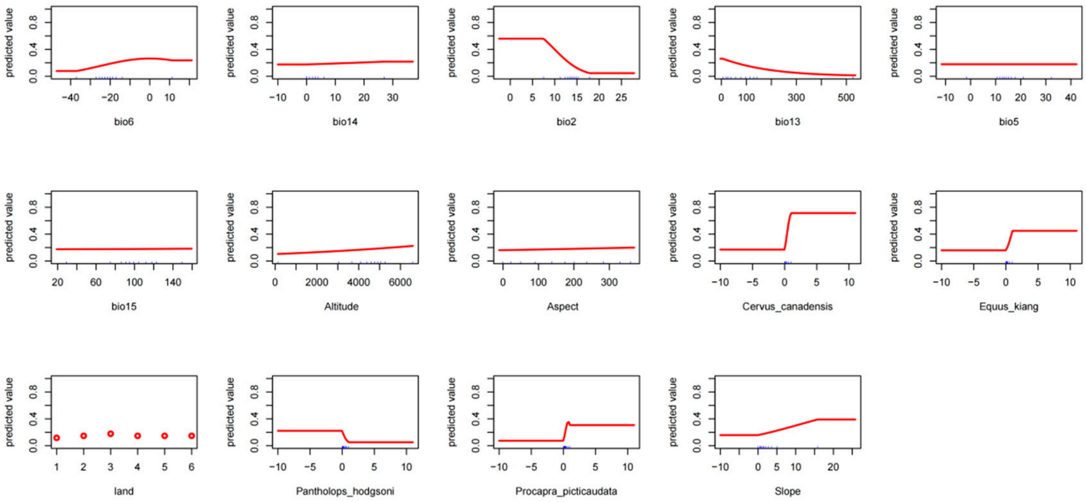

Figure S2-10. Response curves of *Pseudois nayaur* (abotic-biotic)

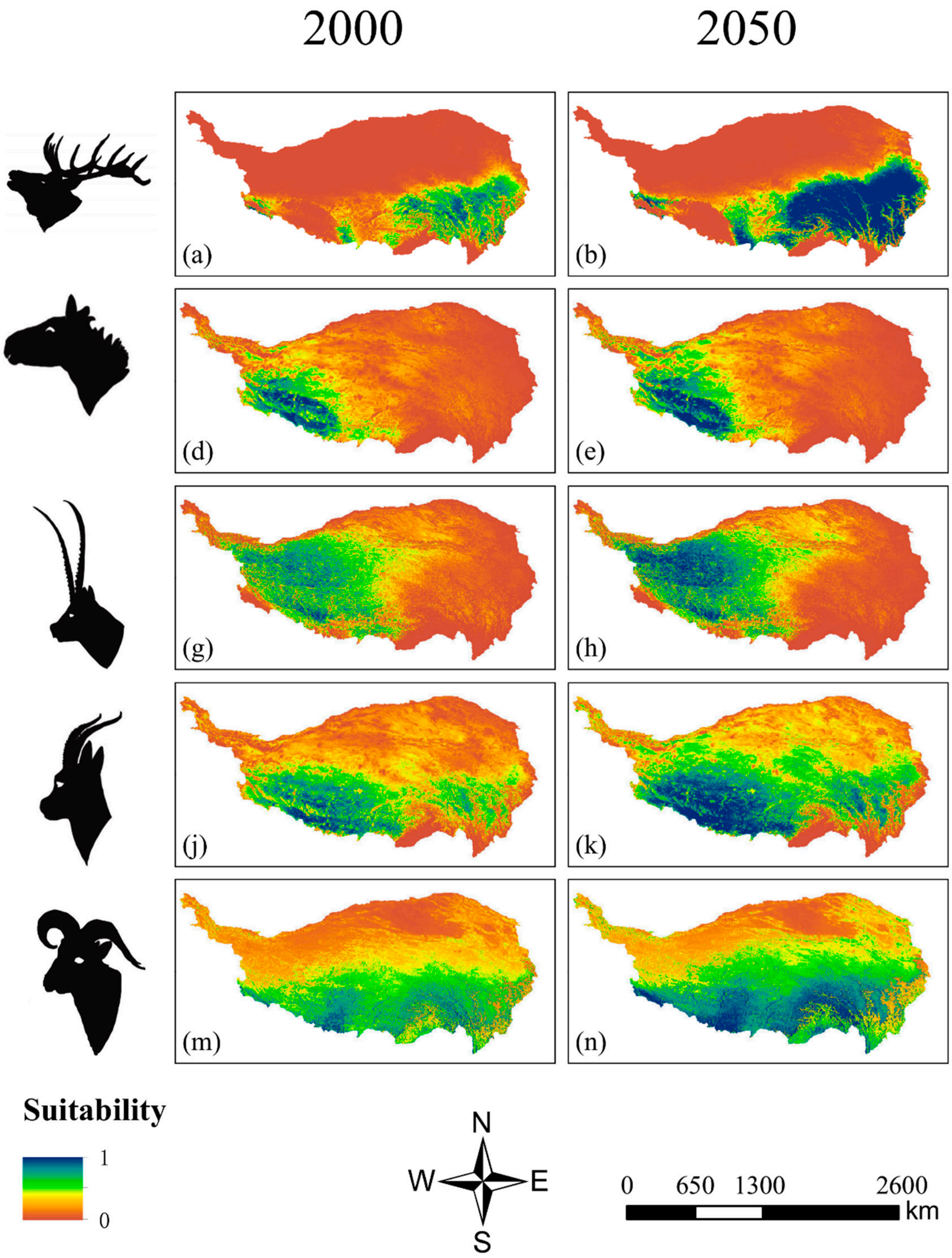

Figure S3. Habitat suitability for each species (abotic): Suitable habitat for each species:(a) Cervus elaphus; (b) Equus kiang; (c) Pantholops hodgsonii; (d) Procapra picticaudata; (e) Pseudois nayaur

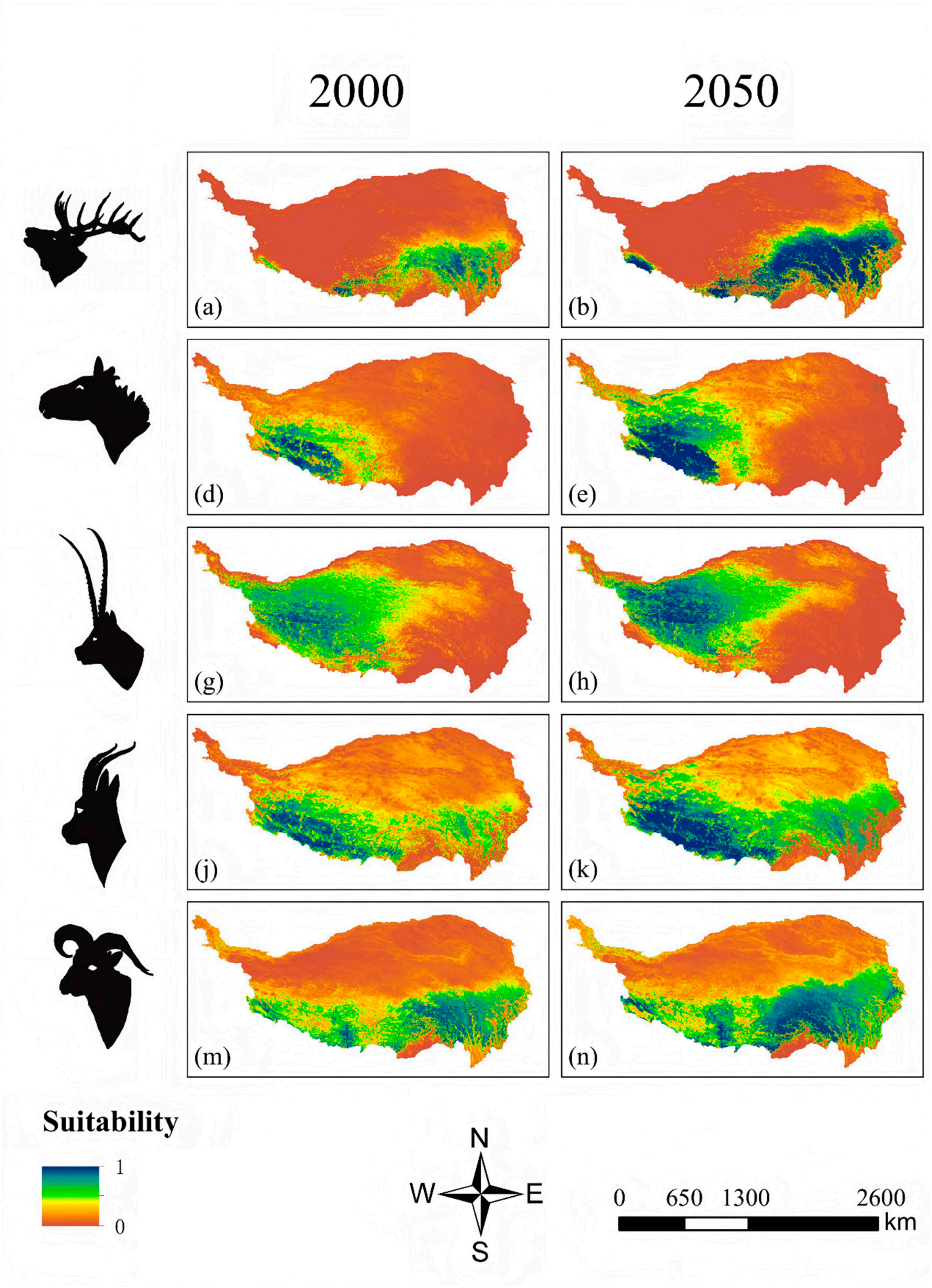

Figure S4. Habitat suitability for each species (abotic-biotic): Suitable habitat for each species: (a) Cervus elaphus; (b) Equus kiang; (c) Pantholops hodgsonii; (d) Procapra picticaudata; (e) Pseudois nayaur
